# Wernicke’s Encephalopathy in an Acute Myeloid Leukemia Patient: A Case Study

**DOI:** 10.4274/tjh.2015.0249

**Published:** 2016-02-17

**Authors:** Muhammet Maden, Gülsüm Pamuk, Yahya Çelik, Ercüment Ünlü

**Affiliations:** 1 Trakya University Faculty of Medicine, Department of Hematology, Edirne, Turkey; 2 Trakya Üniversity Faculty of Medicine, Department of Neurology, Edirne, Turkey; 3 Trakya University Faculty of Medicine, Department of Radiodiagnostics, Edirne, Turkey

**Keywords:** thiamine, Wernicke’s encephalopathy, Acute myeloid leukemia

## TO THE EDITOR

Wernicke’s encephalopathy (WE) is a life-threatening disease with acute onset, resulting from thiamine deficiency. Causes are alcohol intake, malnutrition, gastric bypass surgery, human immunodeficiency virus infection, total parenteral nutrition (TPN), chronic dialysis, and cancer [1]. WE may cause neurological symptoms such as nystagmus, ophthalmoplegia, ataxia, confusion, convulsions, delirium, coma, and acute psychoses [2].

**Figure 1:**
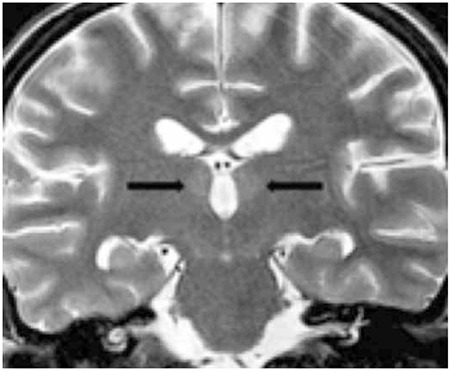
Coronal T2-weighted image shows symmetrical, weak, and limited increased signal intensity in the medial thalami and in the third ventricle-facing surface.

An 18-year-old female patient with refractory acute myeloid leukemia (AML) was given FLAG-IDA (fludarabine, cytosine arabinoside, idarubicin) chemotherapy protocol. As she developed typhlitis, oral intake was stopped; broad-spectrum antibiotics and TPN without any vitamin supplementation were started. She developed a fixed look to a point, chin and upper extremity spasms, and urinary incontinence on the 38th day of chemotherapy. Neurological examination showed non-lateralization, bilateral light reflexes were +/+, verbal stimuli were negative, and the reflex response to painful stimuli was positive. Laboratory results showed hemoglobin of 7.2 g/dL, leukocytes of 3380/mm3, neutrophils of 2890/mm3, platelets of 48,000/mm3, and normal blood biochemistry. The level of serum thiamine could not be measured, because the laboratory did not have the capabilities to measure it. Brain diffusion MRI showed increased signal intensity in the medial thalami (Figure 1). The patient was diagnosed with WE. She was given 1500 mg/day thiamine i.v. for 3 days and 250 mg/day thiamine i.v. for another 5 days. On the fourth day of thiamine infusion, her general condition began to improve and she started giving one-word responses to verbal stimuli. Her convulsions disappeared and she started to form short sentences and walk without assistance; she was discharged on the 30th day of thiamine replacement therapy. Currently, 2 years have passed since the WE and the patient is in complete hematologic remission.

In cancer patients, WE may develop because of certain chemotherapeutic agents (especially doxifluridine, ifosfamide, and 5-fluorouracil) [3], malnutrition, and thiamine depletion due to fast-growing tumor cells or gastrointestinal bypass surgery [2]. In the literature, there are a few cases of WE in AML. The diagnosis of WE in cancer patients is difficult because there are many causes of similar symptoms, such as confusion-causing hypoxia, infections, electrolyte imbalance, opioid medications, chemotherapy, brain and meningeal metastases, and delirium [4]. To make a definite diagnosis of WE, it should be clinically suspected. WE may be verified with the measurement of thiamine concentration in blood or erythrocyte transketolase activity; however, these tests are not widely available [3]. Magnetic resonance imaging (MRI) has 93% specificity and 53% sensitivity to verify the diagnosis [2]. Bilateral dorsomedial thalamus, tectal plaque, and periaqueductal gray matter signal abnormalities are observed as classical in MRI [5]. WE should be treated empirically with 500 mg of thiamine 3 times per day for 2-3 days. If there is no response, supplementation may be discontinued after 2-3 days. In case an effective response is observed, 250 mg of thiamine should be continued daily until clinical improvement [2].

Thiamine supplementation should definitely be added to TPN or the diets of hematologic malignancy patients with poor oral nutrition. When symptoms such as ataxia, confusion, or ophthalmoplegia appear in these patients, brain MRI should be immediately performed and thiamine infusion should be initiated immediately in the case of strong suspicion.

## References

[1] Papila B, Yildiz O, Tural D, Delil S, Hasiloglu ZI, Ayan F, Papila C (2010). Wernicke’s encephalopathy in colon cancer. Case Rep Oncol.

[2] Sechi G, Serra A (2007). Wernicke’s encephalopathy: new clinical settings and recent advances in diagnosis and management. Lancet Neurol.

[3] Basu TK, Dickerson JW (1976). The thiamin status of early cancer patients with particular reference to those with breast and bronchial carcinomas. Oncology.

[4] Kuo SH, Debnam JM, Fuller GN, Groot J (2009). Wernicke’s encephalopathy: an underrecognized and reversible cause of confusional state in cancer patients. Oncology.

[5] Zuccoli G, Siddiqui N, Bailey A, Bartoletti SC (2010). Neuroimaging findings in pediatric Wernicke encephalopathy: a review. Neuroradiology.

